# Zinc and copper supplementation in acute diarrhea in children: a double-blind randomized controlled trial

**DOI:** 10.1186/1741-7015-7-22

**Published:** 2009-05-05

**Authors:** Archana Patel, Michael J Dibley, Manju Mamtani, Neetu Badhoniya, Hemant Kulkarni

**Affiliations:** 1 Lata Medical Research Foundation, Nagpur, India; 2 Indira Gandhi Government Medical College, Nagpur, India; 3 The School of Public Health and the George Institute for International Health, University of Sydney, Sydney, NSW, Australia

## Abstract

**Background:**

Diarrhea causes an estimated 2.5 million child deaths in developing countries each year, 35% of which are due to acute diarrhea. Zinc and copper stores in the body are known to be depleted during acute diarrhea. Our objectives were to evaluate the efficacy of zinc and copper supplementation when given with standard treatment to children with acute watery or bloody diarrhea.

**Methods:**

We conducted a double-blind randomized controlled clinical trial in the Department of Pediatrics at Indira Gandhi Government Medical College Nagpur, India. Eight hundred and eight children aged 6 months to 59 months with acute diarrhea were individually randomized to placebo (Pl), zinc (Zn) only, and zinc and copper (Zn+Cu) together with standard treatment for acute diarrhea.

**Results:**

The mean duration of diarrhea from enrolment and the mean stool weight during hospital stay were 63.7 hours and 940 grams, respectively, and there were no significant differences in the adjusted means across treatment groups. Similarly, the adjusted means of the amount of oral rehydration solution or intravenous fluids used, the proportion of participants with diarrhea more than 7 days from onset, and the severity of diarrhea indicated by more than three episodes of some dehydration or any episode of severe dehydration after enrolment, did not differ across the three groups.

**Conclusion:**

The expected beneficial effects of zinc supplementation for acute diarrhea were not observed. Therapeutic Zn or Zn and Cu supplementation may not have a universal beneficial impact on the duration of acute diarrhea in children.

**Trial registration:**

The study was registered as an International Standard Randomized Controlled Trial (ISRCTN85071383).

## Background

Diarrhea causes an estimated 2.5 million child deaths in developing countries each year, 35% of which are due to acute diarrhea [1]. The importance of zinc and copper in the pathophysiology of acute diarrhea is highlighted by the significantly higher daily fecal losses of these elements during acute diarrheal episodes than the fecal losses in unaffected children [2]. Conversely, zinc supplementation exhibits therapeutic action by facilitating the transport of water and electrolytes across the intestinal mucosa, preventing villous atrophy and improving overall immunity [3]. A pooled analysis of the impact of zinc supplements added to the treatment regimens for acute diarrhea has reported a beneficial effect of reduction in the duration of diarrhea [4]. A recently published Cochrane review has reported a reduction of the duration of acute diarrhea in the zinc group by 12 hours, but there was significant heterogeneity between the trials examined [4,5]. Consequently, the current paradigm favors the use of Zn supplementation for treatment of acute diarrhea in children [6].

The reported efficacy of zinc therapy for acute diarrhea, although encouraging, needs to be considered in the light of several caveats. First, the initial pooled analysis reported a beneficial effect of zinc on duration of diarrhea but a similar effect on the volume of stools was not included in this analysis or the subsequent Cochrane review [4]. Second, additional supplementations in the form of multivitamins or vitamin A were also administered with zinc and placebo in most of the studies included in these reviews, and it is difficult to ignore the possibility of synergistic effects of the co-interventions. Third, the potential beneficial effects of zinc supplementation across different sub-groups of children with acute diarrhea based on age, nutritional status or diarrheal etiology are still not established [7,8]. Lastly, community-based trials are more prone to difficulties with monitoring, compliance and measurement of the trial outcomes such as stool frequency, volume and duration. Large community-based, cluster-randomized, double-masked, placebo-controlled trials of daily prophylactic supplements of 10 mg of zinc found no significant difference in frequency of diarrhea, duration, all-cause hospitalization rates or overall mortality, in Nepal, India and Zanzibar [9-11].

It has been speculated that simultaneous depletion of the copper stores can affect diarrhea morbidity and mortality [12], but the preventive or therapeutic role of copper supplementation is still unclear. It is possible that children supplemented with both zinc and copper experience greater reductions in the duration of diarrhea. We therefore conducted a hospital-based double-blind randomized controlled trial to evaluate the efficacy of zinc and copper supplementation when given with standard treatment to children with acute watery or bloody diarrhea. The primary hypothesis of the study was that the children supplemented with zinc or zinc and copper would experience a reduction in the duration of diarrhea, the volume of stool output and the rates of complications, as compared with those receiving placebo.

## Methods

This was a double-blind, randomized, placebo-controlled clinical trial. The eligibility criteria for this trial were all children aged 6 months to 59 months attending the Indira Gandhi Government Medical College and Hospital in Nagpur, India, with more than three unformed stools in the prior 24 hours; duration of diarrhea up to 72 hours; and ability to accept oral fluids or feeds. Children who were severely dehydrated as per the World Health Organization (WHO) criteria and unable to accept treatment orally were rehydrated with Ringer's lactate and reviewed 4 hours later for ability to take orally [13]. The children were screened for eligibility by a trained study physician. The exclusion criteria were: chronic or severe complicating illness, known positive HIV status, kwashiorkor, residing outside a radius of 30 km around the hospital, participating in another study or already enrolled in this study. The consent procedure was administered to parents or guardians of the children and those who gave informed consent were enrolled and randomized. The Ethics Committee of Indira Gandhi Government Medical College, Nagpur, and the Human Research Ethics Committee of the University of Newcastle, New South Wales, Australia (HREC Approval No: H-500-0203) approved the study protocol, and the treatment effects monitoring committee monitored the trial for safety. The trial is registered with International Standard Randomized Controlled Trial with the unique identifier ISRCTN85071383.

### Randomization and blinding

Each recruited child was sequentially assigned to one of the following three treatment arms using a randomization protocol fixed *a priori *: placebo (Pl) arm, zinc (Zn) only arm, and zinc and copper (Zn+Cu) arm. Single-site, blocked randomization procedure was used for random allocation with blocks of sizes three, six and nine in equal proportions to ensure uniform allocation ratio. The treatment allocation sequence was generated off site by an investigator (HK) not directly involved in the data collection, using the ClinStat software package [14]. The code list of the placebo and the treatment groups was secured and held only by the pharmacist at the Universal Medicaments Pvt. Ltd, Nagpur, until initial data analysis was completed. The bottle packs were sequentially labeled according to the treatment allocation list and assigned to patients by the research physician.

### Interventions

Brown bottles of Pl, Zn and Zn+Cu supplements were prepared by Universal Medicaments Pvt. Ltd, Nagpur, India. The contents were checked by two independent laboratories at the beginning of and during the trial. All the bottles contained a brown-colored syrupy liquid of pH 3 to 4 and weight 1.2 mg/ml, similar in appearance and taste. The Zn supplement bottle had zinc sulfate equivalent to 20 mg/5 ml of elemental zinc, and the Zn+Cu supplement bottle had copper sulfate equivalent to 2 mg/5 ml elemental copper in addition to zinc in the aforementioned dose. The therapeutic dose for all participants was 0.5 ml/kg/day of the syrup (that is, the dose of zinc was 2 mg/kg/day and of copper was 0.2 mg/kg/day). The dose was repeated if the patient vomited following administration. The syrups were administered during the hospital stay and continued after discharge to complete a total duration of 2 weeks from enrolment in the trial. Treatment adherence was measured by weighing the bottles at enrolment, at discharge and on the 14th day of follow-up. In hospital, the patients were monitored for dehydration and fluid balance was maintained using the WHO standard guidelines and oral rehydration solution (ORS) [13]. Briefly, each child was given approximately 100 ml/kg of ORS during the first 4 hours by frequent sips using a spoon, and on-going fecal losses were replaced with the same solution on a volume-to-volume basis until diarrhea ceased. Mothers were encouraged to nurse or feed their children.

### Baseline assessment

At enrolment, the study research physician collected information from the mother about: age, gender, duration of illness (fever, vomiting, diarrhea), degree of dehydration, type of stools, immunization status, existing feeding practices, maternal education, number of children in the household, household assets and facilities, water safety, hand sanitation, intake of anti-diarrheal or antimicrobial agents, type of rehydration practiced at home, and hemoglobin concentration (Hemocue method) [13,15]. The household wealth index, water and hand sanitation scores were composite indices derived by scoring different factors that contributed to these indices using principal component analysis [16]. Factors assessed for the household wealth index were ownership of electricity, radio, television, refrigerator, bicycle, scooter and land, main material used for dwelling floor and fuel used for cooking. For the water safety score the main source of drinking water, water storage and treatment of drinking water before use were assessed, and for the hand sanitation score washing of hands by soap and water, mud, plain water or not washed before feeding the child and after going to latrine were assessed.

Nutritional status was assessed by measuring weight and height using standard methods and calculating weight-for-age and weight-for-height Z scores using the WHO 2005 Anthro software [17]. Weight was measured to nearest 100 gm using an electronic scale (Wedderburn Tanita HD-316). For children aged under 2 years length was measured in the supine position on a wooden sliding board; those over 2 years had stature measured using a height board. Venous blood samples were collected and immediately centrifuged (3500 rpm or G of 1600 for 15 minutes) in metal-free serum gel tubes (Greiner-Bio-One, Austria) and transported to the laboratory for serum ferritin (Micro particle Enzyme Immunoassay), serum zinc, and serum copper estimation using atomic absorption spectrophotometer (Perkin Elmer Model AA 600 with Zeeman background correction, and HGA-600 graphite furnace and an AS- 800 auto sampler were used together with a zinc/copper hallow cathode lamp).

### Monitoring of participants

The patients were monitored for dehydration, vomiting, complications (electrolyte imbalance, hemolytic uremic syndrome, septicemia, co-morbidities such as severe anemia, malaria, pneumonia, meningitis, and death), and the need for unscheduled intravenous (IV) fluids (use of IV fluids on appearance of severe dehydration despite appropriate ORS administration) and its volume (in milliliters) until discharge. Oral rehydration salts and water were supplied in a container of known volume capacity and the total amount consumed from start of the study to the cessation of diarrhea was calculated in milliliters. Stool output was measured every 6 hours by placing the child in a cot with a funnel connected to pre-weighed calibrated plastic collection jars to estimate stool volume (in milliliters) and weight (in grams) by using an electronic scale. Disposable urine collection bags were used with frequent changing to ensure that urine was not mixed with stool to measure urine volume.

After cessation of diarrhea, patients were discharged and instructed to continue the supplementation at home in similar doses, and to return to the hospital for a scheduled follow-up check on the 14th day after discharge, or earlier if there was repeated onset of vomiting or diarrhea or any other illness. They were also advised not to administer any other medication. The weight of syrup bottles was measured at the start of administration, at discharge and at the end of 14 days, to assess treatment adherence in hospital and at home. A second venous blood sample was obtained at a follow-up visit after the completion of 14 days of treatment for assessment of serum zinc and copper.

A child was discontinued from the study for any of the following reasons: complications such as electrolyte imbalance, azotemia, convulsion, acidosis, congestive heart failure, hemolytic uremic syndrome, septicemia, loss of consciousness or death, preventing the child from taking oral fluids or medications; occurrence of a serious adverse event; parent or guardian withdrawal of consent; or if the patient left against medical advice.

### Outcome measures

The following primary outcomes were measured: duration of diarrhea (in hours) from onset and from admission until cessation of diarrhea (passage of soft/formed or no stools for two consecutive 8-hour periods), and the total stool weight in grams during hospitalization. Secondary outcomes included the amount of ORS and the amount of IV fluids used, absolute and percentage change in zinc and copper at day 14 after discharge as compared with baseline in each patient and their means in each group, the rate of complications in hospital, and episodes of any or severe dehydration in hospital.

### Sample size calculation

The sample size was calculated on the basis of the hypothesis requiring the largest number of subjects (for example, 15% reduction in estimated mean duration of diarrhea of 93 ± 43 hours based on unpublished data) and assuming 90% power, a Bonferroni-corrected 5% level of significance (permitting comparisons of two Zn-containing trial arms with the placebo arm) and a 2-tailed test. The required sample size was 234 subjects per group. Allowing for an expected attrition rate of 15%, the required sample size was 808.

### Statistical analysis

Data was entered each day into pre-programmed Microsoft Access software database. Stata 10/IC (Stata Corporation, College Station, TX) software was used for statistical analysis. Anthropometric indicators were calculated using WHO's 2005 Anthro software [17]. Baseline characteristics of the three treatment groups were compared using chi-square tests for categorical variables and ANOVA (or the non-parametric equivalent Kruskal-Wallis test) for continuous variables. Differences between the intervention groups were estimated using multiple regression after adjusting for baseline covariates such as age, gender, prior duration of diarrhea, weight for age less than -2 *Z *-score, dehydration status, any medication received, water safety, wealth index, type of stool, serum zinc and serum copper. Using intention to treat analysis, the unadjusted and adjusted odds ratio (adjusted for the baseline variables) of diarrhea longer than 7 days from onset, of complications in hospital, of more than three episodes of some dehydration and of severe dehydration experienced in hospital was estimated using logistic or Poisson regression models. Cox proportional hazards models were used to estimate the relative hazards (RH) of cessation of diarrhea in the three groups and to explore associations between the same baseline covariates and outcome. The Kaplan-Meier curves for the cumulative probability of cure from diarrhea were also plotted for the three groups and the overall difference in the rates of diarrhea cessation was examined using the log-rank test.

### Role of the funding source

The study sponsor, The Wellcome Trust, played no role in the design or implementation of the collection, analysis, or interpretation of data, or in the writing of this report, or in the decision to submit this paper for publication.

## Results

### Admission characteristics of participants

**Figure 1:**
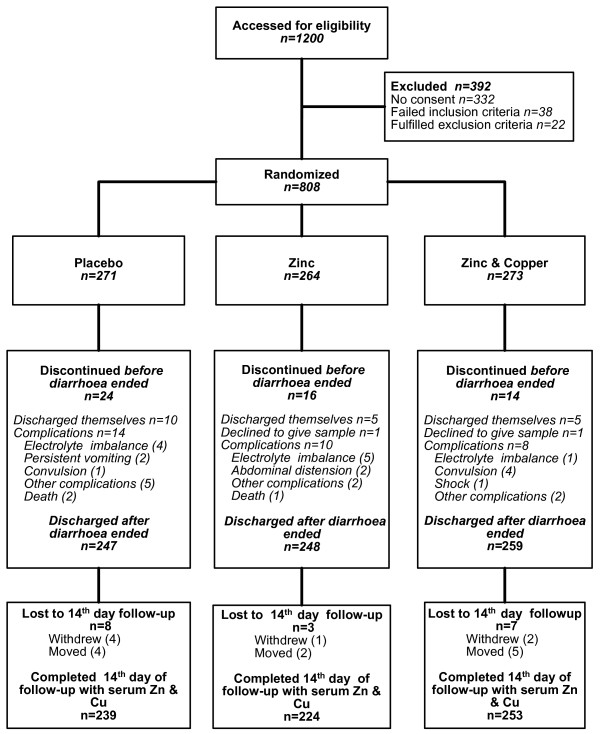
**Flowchart**.

A total of 1200 children were screened from August 2003 to October 2006 for eligibility and 808 were randomized (Figure 1). Each child was followed for 3 months. Imbalance by chance across trial arms was observed in the following baseline characteristics: dehydration status, ORS received by the child prior to enrolment and serum zinc (Table 1). The overall mean duration of diarrhea before enrolment was 35.4 hr and balanced across the groups. There was no significant difference in nutritional status and sanitation indicators across the groups. The proportion of children discharged after cessation of diarrhea was 91%, 94% and 95% in the Pl, Zn and Zn+Cu groups respectively (Figure 1). There were three deaths, two in the Pl group and one in the Zn group. Based on the results of the interim analysis, the treatment effects monitoring committee found no relationship of the deaths to the trial treatments. Serious adverse events related to the syrups were not experienced by any child.

**Table 1:** Comparison of baseline characteristics of the children by groups.

	Placebo (*N *= 271)	Zinc (*N *= 264)	Zinc+Copper (*N *= 273)
Age [months, mean (SD)]	18.01 (11)	17.78 (11.6)	17.80 (10.8)
Age categories [*N *, (%)]			
≤ 12 months	102 (37.6)	119 (45.1)	111 (40.7)
13 to 24 months	119 (43.9)	93 (35.2)	105 (38.5)
> 24 months	50 (18.5)	52 (19.7)	57 (20.9)
Male gender [*N *, (%)]	168 (62)	149 (56.4)	160 (58.6)
Mean number of children in family under 5 years	1.61 (0.8)	1.73 (0.8)	1.72 (0.8)
Mother's education [schooling years, mean (SD)]	6.72 (4.3)	7.46 (4.1)	6.70 (4.3)
Immunization complete to date [*N *, (%)]	178 (65.7)	190 (72)	181 (66.3)
Any breastfeeding [*N *, (%)]	161 (59.1)	142 (53.8)	150 (55)
Duration of illness (hours)	39.41 (22.2)	42.57 (26.9)	42.98 (27.7)
Duration of diarrhea (hours)	35.03 (20.5)	35.80 (20.6)	35.28 (20.3)
Duration of vomiting (hours)	20.92 (21)	20.19 (21.4)	19.93 (19.9)
Duration of fever (hours)	23.96 (24.2)	25.39 (26.3)	24.75 (29)
Dehydration status [*N *, (%)]			
None	200 (73.8)	202 (76.5)	232 (85)
Some	67 (24.7)	57 (21.6)	32 (11.7)
Severe	4 (1.5)	5 (1.9)	9 (3.3)
Dysentery [*N *, (%)]	19 (7.0)	27 (10.0)	32 (11.7)
Received ORS [*N *, (%)]	122 (45.0)	129 (48.9)	104 (38.1)
Received any other medications [*N *, (%)]	127 (46.9)	122 (46.2)	116 (42.4)
Weight-for-age *Z *-score [mean (SD)]	-2.14 (1.2)	-2.07 (1.2)	-2.17 (1.2)
Weight-for-age *Z *-score < -2 [*N *, (%)]	148 (54.6)	133 (50.4)	142 (52)
Weight-for-height *Z *-score < -2 [*N *, (%)]	145 (53.5)	138 (52.3)	148 (54.2)
Height-for-age *Z *-score < -2 [*N *, (%)]	91 (33.6)	78 (29.6)	96 (35.2)
Household wealth index [mean (SD)]	0.01 (1.1)	0.07 (1)	--0.08 (0.9)
Water safety score [mean (SD)]	1.78 (0.9)	1.74 (0.9)	1.90 (1)
Hand washing score [mean (SD)]	1.24 (1)	1.20 (1)	1.25 (1.1)
Serum zinc [μg/dl, mean (SD)]	76.1 (30.2)	71.4 (37.9)	66.1 (28.2)
Serum zinc ≤ 60 μg/dl [*N *, (%)]	86 (31.7)	116 (43.9)	135 (49.5)
Serum Copper [μg/dl, mean (SD)]	122.4 (34.4)	124.3 (39.7)	123.9 (34.8)
Hemoglobin % [g/dl, mean (SD)]	9.60 (1.9)	9.89 (1.7)	9.55 (1.9)

### Treatment adherence

The proportion of children who completed 14 days of oral syrup intake in the Pl, Zn and Zn+Cu groups after discharge was 96.8%, 90.3% and 97.7% respectively (Figure 1). Overall, 66.6% children consumed more than 80% of the prescribed amount of syrup (Table 2). In hospital, more than 80% of the supplements were observed to be consumed in 76.6%, 63.7% and 60.3% of the Pl, Zn and Zn+Cu groups, respectively, and at home in 46.4%, 42.4% and 41.5%, respectively. This level of adherence with the trial treatments corresponded to an average daily intake of 14.3 mg of zinc in the Zn group, and 13.5 mg of zinc and 1.3 mg copper in the Zn+Cu group over 14 days.

**Table 2:** Percentage consumption of supplements by treatment group.

Percentage consumption*	Intervention		
	
	Placebo	Zinc	Zinc+Copper
During stay in hospital (*n *(%))			
0 to 50%	11 (4.1)	30 (11.4)	32 (11.7)
> 50 to 80%	45 (16.6)	59 (22.4)	69 (25.3)
> 80 to 100%	71 (26.2)	58 (22)	53 (19)
100 to 150%	95 (35.1)	77 (29.2)	80 (29.3)
> 150%	18 (6.6)	21 (7.95)	19 (6.96)

Total (mean ± sd)	108.6 ± 84	100.2 ± 68	94 ± 46

At home (*n *(%))			
0 to 50%	47 (17.3)	63 (23.9)	68 (24.9)
> 50 to 80%	81 (29.9)	78 (29.5)	80 (29)
> 80 to 100%	56 (20.7)	36 (13.6)	43 (15.8)
100 to 150%	43 (15.9)	52 (19.7)	50 (18.3)
> 150%	12 (4.43)	16 (6.1)	12 (4.4)
Total (%, mean ± sd)	79.4 ± 37.7	78.6 ± 44	74.9 ± 40.9
	
Intake of zinc per day [mg, mean ± sd ]		14.3+6.9	13.5+5.1

*Percentage consumption = (amount of supplement consumed/amount of supplement prescribed) × 100

### Effect of supplements on study outcomes

The mean duration of diarrhea from enrolment and the mean stool weight during hospital stay were 63.7 hours and 940 g, respectively, and there were no significant differences in their adjusted means across trial groups (Table 3). Similarly, there were no differences in the adjusted means of the amount of ORS or IV fluids used in the three groups. The median duration in hours (IQR) of diarrhea in the Pl, Zn and Zn+Cu group was 48(48), 54(54) and 54(42), respectively. The proportion of patients with diarrhea for more than 3 days was 26.7%, 27.8% and 27% in the Pl, Zn and Zn+Cu groups, respectively. The proportion of patients with diarrhea for more than 5 days was 4.9%, 7.3% and 7.4% in the Pl, Zn and Zn+Cu groups, respectively. There was no significant difference in diarrhea longer than 14 days. The observed reduction in the point estimates of the severity of diarrhea indicated by rates of complications, more than three episodes of some dehydration or any episode of severe dehydration after enrolment were not statistically significant (Table 3).

**Table 3:** Study outcomes by intervention.

Outcome variable	Intervention	Mean ± sd	Difference (95%CI)*	Adjusted Difference (95%CI)**
Stool weight (g)				
	Placebo	876.9 ± 1194.1		
	Zn	972.3 ± 920.2	95.4 (-85.4,276.2)	21.1 (-40.8,83)
	Zn+Cu	972.3 ± 1210	94.5 (-86.7,275.7)	23.8 (-35.5,83.1)
Duration of diarrhea (hours)^a ^from enrollment				
	Placebo	62.2 ± 33.5		
	Zn	64.4 ± 37.8	2.2 (-4.1,8.5)	1.9 (-2.23,2.55)
	Zn+Cu	64.4 ± 35	2.2 (-3.8,8.2)	-0.9 (-2.37,2.2)
Amount of ORS used in hospital (ml)				
	Placebo	1666.5 ± 1372.7		
	Zn	1837.1 ± 1719.4	170.6 (-93.3,434.5)	42.8 (-45.6,131.2)
	Zn+Cu	1811.9 ± 1499	145.4 (-96.7,387.5)	67.2 (-17.5,152.1)
Amount of IV fluids used in hospital (ml)				
	Placebo	127.3 ± 412.9		
	Zn	127.9 ± 358.2	0.7 (-65,66.4)	4.1 (-16,24.1)
	Zn+Cu	131.7 ± 428	4.5 (-66.4,73.3)	-1.2 (-20.7,18.3)
14th day zinc levels (μg/dL)				
	Pl	76.3 ± 32.9		
	Zn	76.6 ± 35.	0.3 (-5.8, 6.4)	-1.1 (-3,0.9)
	Zn+Cu	74.3 ± 40	-2 (-8.5, 4.6)	-2.3 (-4.1,-0.5)
14th day copper levels (μg/dL)				
	Pl	115.2 ± 30.8		
	Zn	121.2 ± 35.	6.0 (0.1,11.9)	1.7 (-0.4,3.7)
	Zn+Cu	124.6 ± 36.8	9.4 (3.4,15.5)	2.4 (0.5,4.3)
Mean absolute difference from baseline in Zn^c^				
	Placebo	7.2 ± 379.6		
	Zn	59.2 ± 428.5	52 (-20.7,124.7)	34.8 (-11.7,81.2)
	Zn+Cu	81.8 ± 452.8	74.5 (-0.11,149)	71.4 (30.9,111.9)
Mean absolute difference from baseline in Cu^d^				
	Placebo	-79.4 ± 429.2		
	Zinc	-41.2 ± 418.8	38.2 (-37.8,114.3)	14 (-1.5,9.5)
	Zn+Cu	15.6 ± 439.8	95 (39.3,17.7)	19.6 (3.6,35.6)

		*N *(%)	Odds ratio(95%CI)	Adjusted odds ratio(95%CI)

Proportion with diarrhea of > 7 days from onset^b^				
	Placebo	13(5.3)		
	Zn	20(8.1)	1.6 (0.77,3.25)	1.8 (0.78,3.97)
	Zn+Cu	15(5.8)	1.11 (0.52,2.38)	1.18 (0.51,2.73)

		*N *(%)	IRR (95%CI)^$^	Adjusted IRR (95%CI)

Rates of complications in hospital				
	Placebo	14 (5.2)		
	Zn	10 (3.7)	0.73 (0.33,1.65)	0.76 (0.32,1.84)e
	Zn+Cu	8 (2.9)	0.57 (0.24,1.35)	0.68 (0.26,1.78)
More than three episodes of some dehydration in hospital				
	Placebo	6 (2.2)		
	Zn	2 (0.8)	0.34 (0.07,1.7)e	0.35 (0.07,1.77)
	Zn+Cu	5 (1.8)	0.83 (0.25,2.71)	0.94 (0.27,3.34)
Severe dehydration experience in hospital				
	Placebo	12 (4.4)		
	Zn	6 (2.3)	0.51 (0.19,1.37)	0.55 (0.21,1.49)
	Zn+Cu	8 (2.9)	0.66 (0.27,1.62)	0.77 (0.31,1.94)

* The difference is as compared with the placebo group.

**Adjusted for following covariates: age, gender, prior duration of diarrhea, weight-for-age *Z *-score at most -2, dehydration status, receipt of medication, water safety, wealth index, type of stool, baseline serum zinc and serum copper.

^$ ^Incidence rate ratios on Poisson regression. Beneficial effect is < 1.

^a^Censored duration of diarrhea: 16 in Zinc, 14 in Zinc/Copper treatment,24 in Placebo.

^b^Censored proportion with diarrhea > 7 days from onset: 16 in Zinc treatment, 14 in Zinc/Copper treatment, 24 in Placebo.

^c ^Censored 14th day serum Zinc: 32 in Zinc treatment, 19 in Zinc/Copper treatment,21 in Placebo.

^d^Censored 14th day serum copper: 32 in Zinc treatment, 19 in Zinc/Copper treatment,21 in Placebo.

**Figure 2:**
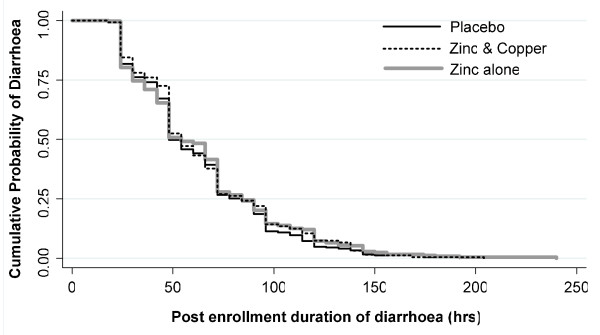
**The Kaplan-Meier curves for post enrolment duration of diarrhea in three groups**.

The Kaplan-Meier curves (Figure 2) for post-enrolment duration of diarrhea showed there was no significant difference in cessation of diarrhea among the three groups (log-rank test *p *= 0.6399). The RH of the Cox proportional hazards model showed no significant reduction in the duration of diarrhea in either Zn or Zn+Cu group after adjusting for baseline covariates. Baseline serum zinc and serum copper had no effect on the duration or volume of diarrhea.

### Effect of supplements on serum zinc and copper levels

At baseline the mean serum zinc concentration in the placebo group was significantly higher than the other treatment groups and it had the least number of children with levels under 60 μg/dl (Table 1). However, the increase in zinc concentrations compared with baseline was significantly (*p *< 0.001) greater in the supplemented groups as compared with the placebo group (Table 3). The mean percentage change in serum zinc from baseline was 17 ± 96, 28.9 ± 86.7 and 34.3 ± 107.7 in the Pl, Zn and Zn+Cu groups, respectively. The mean serum copper at enrolment was similar across treatment groups, and by the 14th day the unadjusted increase in copper concentration was significantly greater in the copper-supplemented group compared with the Pl or Zn groups, and significantly decreased from baseline in the Pl group.

## Discussion

The duration of diarrhea and the total stool output during an episode of acute diarrhea were not significantly different in the supplemented and placebo groups. Other outcome measures such as the use of ORS, IV fluids, episodes of dehydration experienced in the hospital, rates of complications and diarrheal duration more than 7 days were also no different in the children who received Zn or Zn+Cu supplementation as compared with the placebo, when adjusted for nutritional status, type of stool (presence or absence of dysentery) and baseline serum zinc or copper levels. All children received similar doses, frequency and duration of syrups of identical appearance for 14 days and the treatment adherence was similar in the three groups. Serious adverse events related to the syrups were not experienced by any child, suggesting that they can be used safely in treatment protocols.

There could be many reasons for lack of effect observed in our study. We first considered the possibility of either low dose of zinc (an average intake per day of 13.9 mg over 14 days in any of the zinc groups) as compared with other therapeutic studies that have used a fixed dose of 20 mg, or poor treatment adherence, or failure of supplements to replenish the zinc loss, as possible causes of the failure of a favorable response to treatment. However, studies that report administration of fixed dose of 20 mg have not reported the actual mean consumption. Also, in our study there was no difference in treatment adherence across the three groups in the hospital stay or at home after discharge (Table 2). The mean of absolute difference of serum zinc from baseline to the 14th day increased significantly in zinc-supplemented groups, indicating appropriate dose and bioavailability of the supplements (Table 3).

It has been observed that there is a differential extent of zinc deficiency, considered usually as serum zinc less than 60 μg/dl, across study populations, which could explain the variation in effects of either prophylactic or therapeutic zinc supplementation acute diarrhea [7]. It is expected that those countries at high risk of zinc deficiency, that is, prevalence stunting exceeding 20% and estimated prevalence of inadequate zinc intake of more than 25%, would most likely benefit from prophylactic and therapeutic zinc supplementation [18]. However, there is heterogeneity in the effect of zinc during acute diarrhea even in those with low plasma or serum zinc levels. Large studies from Nepal, Zanzibar and India of prophylactic zinc supplementation found no difference in morbidity and mortality of children aged 1 to 48 months between placebo and supplemented groups [10,11,19]. However, a smaller prophylactic study from Bangladesh did observe reduced mortality in children, although the majority of the deaths were pneumonia related [20]. The mean baseline plasma zinc in these studies ranged from 62 to 78.7 μg/dl. A therapeutic study from Bangladesh in infants also reported no effect of zinc supplementation and the mean baseline zinc of the study population was 68.5 μg/dl (see [7]).

Also, not all therapeutic studies that reported a beneficial effect of zinc in acute diarrhea were in children with zinc deficiency. Their serum or plasma zinc ranged from 58.0 to 92.9 μg/dl at baseline, with range of percentage change at the end of the therapy being -1.7 to 42, thus failing to establish a clear relation of response to zinc therapy and underlying low blood zinc levels [10,19,21-26]. By comparison, in our study the mean baseline serum zinc level was 71.2 μg/dl, with the highest proportion of children with serum zinc at least 60 μg/dl in the Pl group. Although the study outcomes were adjusted for baseline serum zinc and copper, it is possible that there was a risk of longer duration of diarrhea in the intervention groups as they had lower baseline zinc. This may perhaps reduce the observed difference in the diarrheal duration between intervention and placebo groups. However, the overall underweight rate in the study population (weight for age less than -2 *Z *-score) was 52.4% and was balanced across the groups. A limitation of this study, common to many existing studies of therapeutic and prophylactic zinc supplementation, is that serum zinc concentrations are not a reliable measure of body zinc status [27]. Instead, measurement of dietary zinc or copper intake and tissue zinc or copper status could perhaps explain the differential impact of zinc with respect to varying tissue zinc status.

Age can modify the beneficial effect of Zn supplementation. Two studies with infants younger than 6 months, included in the Cochrane review, showed no impact of zinc on duration of acute diarrhea. Although tissue zinc status was not measured in these studies, they speculated that perhaps the lack of effect was due to adequate zinc stores acquired *in utero *, through breast feeding and lack of preceding zinc depleting illness [7,28]. However, five studies that included children aged 2 to 36 months (mean age was 14.4 months), a large proportion of which were also ever breast fed reported a beneficial effect [21,22,29-31]. Thus, there is inconsistency in the evidence on whether zinc stores *in utero *or those acquired from breast feeding would diminish the impact of supplementation on duration of acute diarrhea. In our study, 41% of the children were between 6 and 12 months of age, the mean age was 17.9 months and 56% had received any breast feeding.

The impact of zinc supplementation may also be related to different diarrheal etiology at different age groups and study populations. Zinc has been postulated to have less impact on rotavirus diarrhea and the secretory effect of *Escherichia coli *heat-stable enterotoxin but beneficial effects on enteropathogenic *E. coli *infection in animal models [32-35]. The heterogeneity in effects of zinc for acute childhood diarrhea could also be due to a varied patient population, study designs (field based or hospital based), the methods of monitoring diarrhea and the outcome measured. In a pooled analysis of three data sets, the Zinc Investigators Collaborative Group in 1999 [36] reported a multivariate RH of 0.85 (0.78, 0.92) or a 15% lower probability for continuation of acute diarrhea in the zinc group in studies from Indonesia (*n *= 1,368), India (*n *= 931), and Bangladesh (*n *= 101). Although this contrasts with our study, two of the three studies (Indonesia and Bangladesh) showed no difference with RH (95% CI) for continuation of diarrhea of 0.92 (0.83, 1.02) and 0.85 (0.57, 1.28), respectively. The pre-enrolment diarrheal duration for Indonesia, India and Bangladesh was 1.9, 2.7, and 3.4 days respectively, and the mean post-enrolment diarrheal duration was 3.5, 5.1 and 4.5 days, respectively. Only the Indian study showed a mean difference of 1 day from placebo with an effect size of 0.24 (95% CI 0.10, 0.37) in the duration of diarrhea. Further, the Indian and Indonesian studies were community-based trials where cessation of diarrhea was defined as a 48-hour period free of diarrhea (three to four unformed stools in one day) measured from a 5 to 7 day recall of the mother. The Bangladesh trial was a hospital-based study which recruited children with diarrhea for less than 72 hours, monitored 8-hourly in the hospitals for stool output and cessation of diarrhea defined as passage of a soft formed stool. These different study designs indicate that separate meta-analyses for hospital-monitored and community-based studies are required to fully assess the therapeutic effects of zinc in acute diarrhea.

Finally, in several studies it is difficult to ignore the interactions of zinc with multivitamins or co-interventions administered to the study populations. The Zinc Investigators Collaborative Group further conducted a meta-analysis of three of the above mentioned studies and two additional studies [25,37] from India and Bangladesh. Since multivitamins including vitamin A were administered with zinc in three studies, a potentially beneficial interaction of zinc with vitamin A or other vitamins cannot be ruled out. Zinc is involved in the release of vitamin A from liver cells and in the synthesis of retinol binding protein, and the effect of zinc in infectious diseases may be dependent on an adequate vitamin A status [38-40]. Moreover, there are no studies directly examining the relationship between the different B vitamins and diarrheal disease, which makes it difficult to arrive at any conclusions about their effects on this outcome [30]. It is also noteworthy that blinding is a challenge when combinations of drugs are used. In one of the Indian studies included in a recent meta-analysis, glucose water was used as a placebo which is neither identical in appearance or taste to the intervention, making the double blinding questionable [25].

This trial is the first to evaluate the impact of oral zinc and copper administration on duration of acute diarrhea, serum zinc and copper levels after 14 days of supplementation. Large oral doses and a relatively low dose of dietary zinc are known to interfere with copper bioavailability [41], and contrarily, copper may interfere with zinc absorption and obliterate its effect [42]. A diarrheal morbidity trial of 4 months zinc supplementation in north Indian children reported a significantly lower copper level in the zinc supplemented group, and an adverse effect on copper levels at the end of the study [43]. However, we supplemented zinc and copper in the same ratio as in a customary diet and therefore this Zn-Cu interaction would be unlikely [44,45]. Also, a significant fall in serum copper levels from baseline was observed only in the placebo group, which had no effect on diarrheal duration. Similar results have been observed in other studies [11,20,21].

## Conclusion

The results of our study further highlight the heterogeneity of results of zinc supplementation in acute diarrhea. Thus, it appears that therapeutic Zn supplementation may not have a universal beneficial impact on the duration of acute diarrhea. A re-examination of all available trial results, including this trial, is needed to dissect out the potential contributors to heterogeneity of trial results before Zn can be universally recommended for treatment of acute childhood diarrhea.

## Abbreviations

IV: Intravenous; ORS: Oral rehydration solution; Pl: Placebo; RH: Relative hazards; WHO: World Health Organization; Zn: Zinc; Zn+Cu: Zinc and copper.

## Competing interests

The authors declare that they have no competing interests.

## Authors' contributions

AP developed the study protocol, questionnaires and clinical trial procedures, directed the conduct of the trial, held routine meetings with the ward medical and the field staff, contributed to the quality control, data cleaning, data analysis, data interpretation and wrote the first draft of the paper. MJD was the co-investigator from Australia, who contributed to the development of the protocol, helped in the development of study questionnaires and trial procedures, contributed to the data analysis, data interpretation and edited the paper. MM assisted with drafting the manuscript, and NB helped with data analysis. HK generated the block randomization list, assisted with data analysis and editing of the paper. All authors contributed to the design and implementation of the study, reviewed drafts of the manuscript, have read and approved the final draft.

## Pre-publication history

The pre-publication history for this paper can be accessed here:


